# Optimisation of External Factors for the Growth of *Francisella novicida* within *Dictyostelium discoideum*

**DOI:** 10.1155/2020/6826983

**Published:** 2020-01-20

**Authors:** Ina Kelava, Valentina Marecic, Petra Fucak, Elena Ivek, Dominik Kolaric, Mateja Ozanic, Mirna Mihelcic, Marina Santic

**Affiliations:** Department of Microbiology and Parasitology, Faculty of Medicine, University of Rijeka, Rijeka, Croatia

## Abstract

The amoeba *Dictyostelium discoideum* has been used as a model organism to study host-pathogen interaction in many intracellular bacteria. *Francisella tularensis* is a Gram-negative, highly infectious bacterium that causes the zoonotic disease tularemia. The bacterium is able to replicate in different phagocytic and nonphagocytic cells including mammalian, amoebae, and arthropod cells. The aim of this study was to determine the optimal temperature and infection dose in the interaction of *Francisella novicida* with *D. discoideum* in order to establish a model of *Francisella* infection in the social amoeba. The amoeba cells were infected with a different multiplicity of infection (5, 10, and 100) and incubated at different temperatures (22, 25, 27, 30, and 37°C). The number of intracellular bacteria within *D. discoideum*, as well as cytotoxicity, was determined at 2, 4, 24, 48, and 72 hours after infection. Our results showed that the optimal temperature for *Francisella* intracellular replication within amoeba is 30°C with the MOI of 10. We can conclude that this MOI and temperature induced the optimal growth of bacteria in *Dictyostelium* with low cytotoxicity.

## 1. Introduction

*Francisella tularensis* is a small Gram-negative bacterium that causes the zoonotic disease tularemia. Type A is designated as a category A select agent due to its low infectious dose [1, 2]. There are three subspecies of *F. tularensis* that cause human illness: *tularensis* (Type A), *holartica* (Type B), and *mediasiatica* [3]. *F. novicida*, *F. philomiragia*, and *F. hispaniensis* can cause tularemia in immunocompromised people [4]. *F. noatunensis* has been recognized as a fish pathogen, while recently new environmentally adapted species have been discovered including *F. piscidida*, *F. guangzhouensis*, *F. opportunistica*, *F. Salina*, *F. uliginis*, *F. frigiditurris*, and *F. adeliensis* [4–7].

*Francisella* is known as a fastidious organism with many growth requirements *in vitro*. The bacterium is difficult to culture and grows slowly at 37°C, requiring enriched growth medium [8]. Each species of the genus *Francisella* require different growth and medium conditions. *Francisella* requires medium supplemented with cysteine for cultivation. The bacterium grows on Buffered Charcoal-Yeast Extract Agar (BCYE), Chocolate Agar, Thayer–Martin Agar, and on the media containing hemoglobin, such as Cysteine Heart Agar Base (CHAB). Bacterial growth on blood agar is very slow with a narrow zone of alpha-hemolysis. *Francisella* can also be cultivated in a liquid medium; however it shows a lower replication rate. For example, the most used is Mueller–Hinton medium (MHM) in which bacteria efficiently replicates after incubation of 7 to 10 days. *Francisella* species can be incubated with 5% CO_2_, but it only enhances the growth of *F. tularensis* subsp*. holarctica* LVS (Live Vaccine Strain). *F. novicida*, *F. noatunensis*, and *F. philomiragia* require less nutritive supplements in the medium for *in vitro* growth, possibly due to their adaptation to environmental conditions [8, 9].

Further, the optimal temperature for the growth of *F. tularensis* is 37°C. *F. tularensis* grows slowly at room temperature, in contrast to *F. novicida* and *F. philomiragia*, which survive at 25°C. Because of the complex growth requirement of *Francisella*, most of the human tularemia cases are misdiagnosed by serology or clinical features [10, 11]. Importantly, previous studies demonstrated the impact of temperature and growth medium on the virulence of bacteria and vaccine design [12].

The exact reservoir of *Francisella* spp. in the environment has not been definitely determined. However, bacteria have been strongly associated with water environments [13]. It is assumed that the bacterial existence in the aquatic environment is connected with the ability of *Francisella* to survive and replicate within amoeba cells [14]. Our and other previous *in vitro* studies showed the survival and replication of *F. novicida* in *H. vermiformis* and *A. castellanii*, showing the possible importance of protozoa in *Francisella* ecology [15–17].

However, due to limited tools and antigens to study intracellular lifestyle in free-living amoeba, such as *A. castellanii* and *H. vermiformis*, *D. discoideum* has been used as a model organism to study phagocytosis, cell motility, and virulence factors for many bacterial pathogens, such as *Pseudomonas*, *Legionella*, *Mycobacterium*, *Salmonella*, and *Klebsiella* [18–23]. *Dictyostelium* has been established as a model organism for studying the life cycle of the fish pathogen, *F. noatunensis*, but it has not been established for studying the strains of *Francisella* that cause the disease in humans [24–26]. It has been shown that *Salmonella* requires O-antigen and the type VI secretion system (T6SS) for the survival within amoebae [22]. *Francisella* uses the T6SS to avoid lysosomal fusion within the macrophages [27]. After establishing this model of *F. novicida* infection in *Dictyostelium* it would be interesting to investigate the role of T6SS in this social amoeba for prolonged survival of *Francisella* in nature.

Although the previous studies demonstrated the survival and replication of *Francisella* within macrophages and various cell types, little is known about the adaptation of *Francisella* to protozoa cells [28, 29]. Our study was focused on establishing the optimal external factors required for survival and replication of *F. novicida* within *D. discoideum*. We examined the role of various incubation temperatures and the dose of infection on *F. novicida* capability to survive and replicate within *D. discoideum*.

## 2. Materials and Methods

### 2.1. The Cell Strains and Growth Conditions

The wild type strain of *F. novicida* was kindly obtained from prof. Anders Sjöstedt (Umeå University, Umeå, Sweden)*. F. novicida* was cultured on buffered charcoal-yeast extract (BCYE) agar (Sigma, USA) at 37°C with 5% CO_2_ atmosphere for 24 h. *D. discoideum* strain (AX2) was obtained from prof. Michael Steinert (Technische Universitat Braunschweig, Germany). *D. discoideum* cells were grown in HL5 medium at 25°C. HL5 medium contains peptone (Biolife, Italy), yeast extract (Oxoid, UK), glucose (Merck, Germany), monopotassium phosphate (Kemika, Croatia), and disodium phosphate (Kemika, Croatia).

### 2.2. Cultivation of the Bacteria at Different Temperatures

To determine the survival of *F. novicida in vitro* at different growing temperatures, the bacterial suspensions (10^3^ CFU·mL^−1^) were inoculated in tubes with 50 mL of the buffered yeast extract (BYE) broth (Sigma, USA). The suspensions were incubated at 22, 25, 27, 30, 37°C, and 42°C for 5 days. Every 24 hours, the number of *F. novicida* was determined by plating the serial dilutions on BCYE agar at 37°C.

### 2.3. Infection of Cells

The number of *D. discoideum* (10^5^ amoebae·mL^−1^) was counted using a Neubauer chamber (Thermo Fisher Scientific, USA). The cells were seeded in 96-well plates, incubated overnight and infected with *F. novicida* at a multiplicity of infection (MOI) 5, 10, or 100. For infection, the HL5 medium was diluted with a phosphate buffer (1 : 1). In order to achieve synchronized infection, the cells were centrifuged immediately after infection at 240 *g* for 3 minutes at room temperature. The cells were then incubated at 27°C for 1 hour and washed 3 times with PBS to remove extracellular bacteria. This was considered as a time point zero. At each time point after infection (2, 4, 24, 48, and 72 h), the cells were treated with 0.9% Triton X-100 (Sigma, USA) for 10 minutes to lyse the cells. The number of intracellular bacteria was determined by plating the serial dilutions on BCYE agar.

### 2.4. Growth of Bacteria within Amoeba at Different Temperatures

To determine the influence of different incubation temperatures on the growth of bacteria within amoeba cells, the cells were infected with MOI 10 as described previously. The infected cells were incubated at 22, 25, 27, 30, or 37 °C and the number of intracellular bacteria was determined by plating the serial dilutions on BCYE agar.

### 2.5. Released Lactate Dehydrogenase (LDH) Assay

*D. discoideum* cells were infected with MOI 5, 10, or 100, washed 3 times and incubated for 1 hour (time point zero). At 2, 4, 24, 48, and 72 h after infection, 50 *μ*L of culture supernatant was transferred to a 96-well plate for monitoring cytotoxicity. The CytoTox 96 Non-Radioactive Cytotoxicity Assay (Promega Corporation, USA) was used to quantitatively measure lactate dehydrogenase (LDH) according to the manufacturer's instructions. The absorbance signal was measured at 490 nm using a microplate reader (Tecan Systems, USA).

### 2.6. Statistics

Statistical significances were determined using two-tailed Student's *t*-test. Statistical analyses were performed using Statistica (Statsoft) software version 12 or with Graph Pad Prism version 6.0 software. *p* value < 0.05 were accepted as significantly different and were denoted by ^*∗*^. Exact *p* values are listed in the results.

## 3. Results

### 3.1. Prolonged Survival of Bacteria *In Vitro* Is Temperature Dependent

The effect of different incubation temperatures on the survival of *F. novicida in vitro* was examined. The bacterial suspensions were incubated at temperatures of 22°C, 25°C, 27°C, 30°C, 37°C, or 42°C for 5 days. By every 24 hours, the number of *F. novicida* was determined by plating the serial dilutions on BCYE agar at 37°C.

**Figure 1:**
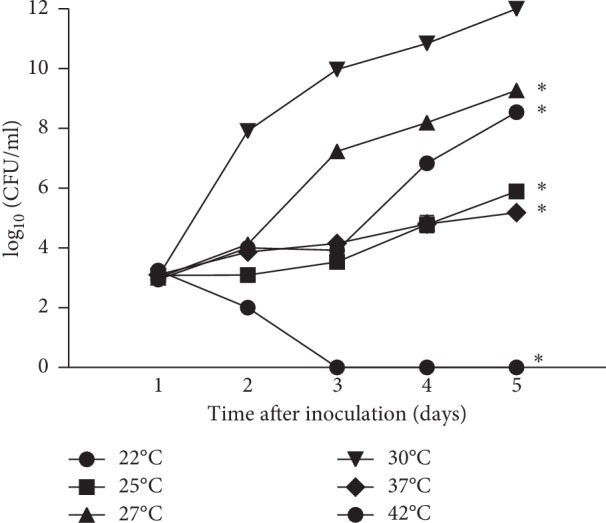
Growth kinetics of *F. novicida* after exposure to different temperatures. The bacterial suspensions were incubated at 22, 25, 27, 30, 37, or 42°C for 5 days. The number of *F. novicida* was determined daily by plating the serial dilutions on BCYE agar at 37°C. The experiments were performed in triplicate and the error bars represent standard deviations. ^*∗*^*p* < 0.05.

Our results show that *F. novicida* replicates with prolonged time of incubation at 22, 25, 27, 30, and 37°C. By day 5, the number of bacteria increased up to 8.0 × 10^5^ CFU·mL^−1^ after incubation at 25°C. At 37°C the number of bacteria increased up to 1.5 × 10^5^ CFU·mL^−1^, and at 22°C up to 8.0 × 10^6^ CFU·mL^−1^, while the incubation temperatures of 27 and 30°C resulted in a higher replication of bacteria, 2.0 × 10^9^ CFU·mL^−1^ and 5.0 × 10^12^ CFU·mL^−1^, respectively. In contrast, when the incubation temperature was 42°C, *F. novicida* was not able to survive after the second day of incubation (Figure 1).

We can conclude that the optimal temperature for bacterial replication *in vitro* is 30°C. In comparison with optimal temperatures, the number of bacteria observed after incubation at 22°C (day 2: *p*=0.003, day 3: *p*=0.003, day 4: *p*=0.028, day 5: *p* < 0.001), 25°C (day 2: *p*=0.002, day 3: *p*=0.002, day 4: *p*=0.012, day 5: *p* < 0.001), 27°C (day 2: *p*=0.002, day 3: *p*=0.002, day 4: *p*=0.012, day 5: *p* < 0.001), 37°C (day 2: *p*=0.003, day 3: *p*=0.003, day 4: *p*=0.011, day 5: *p* < 0.001), and 42°C (day 2, day 3, day 4 and day 5: *p* < 0.001) was significantly different.

### 3.2. Bacterial Growth in Amoeba after Different Dose of Infection

The influence of a different dose of infection on the survival and replication of bacteria within *D. discoideum* was investigated. The amoeba cells were infected with *F. novicida* with MOI 5, 10, or 100. The infected amoeba cells were incubated at 27°C. At 2, 4, 24, 48, and 72 hours after infection, the number of intracellular bacteria was determined by plating the serial dilutions on BCYE agar.

**Figure 2:**
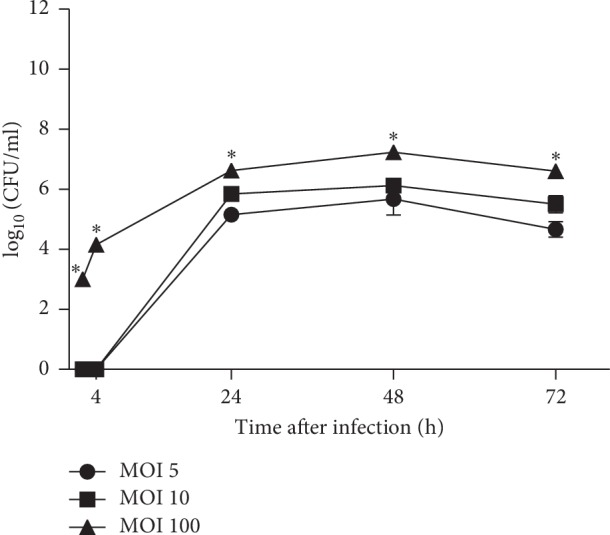
The intracellular growth kinetics of *F. novicida* within *D. discoideum*. The *D. discoideum cells* were infected with *F. novicida* at MOI 5, 10, and 100. At 2, 4, 24, 48, and 72 h after infection, the cells were lysed and suspension was plated on BCYE agar to determine the number of intracellular bacteria. The experiments were performed in triplicate and the error bars represent standard deviations. ^*∗*^*p* < 0.05.

Our results show that at 2 and 4 hours after infection of the amoeba with MOI 5 and 10, *F. novicida* did not replicate within *Dictyostelium*. In contrast, after infection of amoeba cells with MOI 100, bacteria started proliferation after 2 hours of infection and increased up to 10^6^ CFU·mL^−1^ by 24 hours after infection (Figure 2). Each MOI induced replication of bacteria in *Dictyostelium* up to 24 hours after infection. The linear growth was followed by the stationary phase, in which the number of bacteria remained constant among 24 and 48 hours after infection. During the stationary bacterial phase, the number of intracellular bacteria was similar to the dose of infection of 5 and 10 (10^6^ CFU·mL^−1^) (Figure 2). However, the number of intracellular bacteria was higher at the stationary bacterial phase after the infection of the cells with MOI 100 (10^7^ CFU·mL^−1^) (Figure 2). At 72 hours after infection, the intracellular number of bacteria decreased to 10^4^ CFU·mL^−1^ with the dose of infection 5, to 10^5^ CFU·mL^−1^ with the dose of infection 10, and to 10^6^ CFU·mL^−1^ with the dose of infection 100 (Figure 2).

Our results showed that the number of intracellular bacteria was statistically different after infection of amoeba cells with MOI 10 and MOI 100 (*p*=0.002) but not with MOI 5 and MOI 10 (*p*=0.09). The number of intracellular bacteria after infection of *Dictyostelium* cells with MOI 5 and MOI 100 was statistically different at all observed time points (*p* < 0.001).

We can conclude that the infection of amoeba cells with MOI 100 induced the highest intracellular growth of *F. novicida* in *Dictyostelium*. The number of intracellular bacteria within *D. discoideum* increased with higher MOI and prolonged incubation times.

### 3.3. The Induction of Cytopathogenicity in Amoeba Cells after Infection with Different MOI

We examined the ability of the *F. novicida* to induce a cytopathogenic response in *D. discoideum* cells. After infection of the amoeba cells with MOI 5, 10, or 100, the cells were washed 3 times and incubated for 1 hour. The supernatants of the infected cells were sampled at 2, 4, 24, 48, and 72 hours after infection and examined for the presence of the LDH.

**Figure 3:**
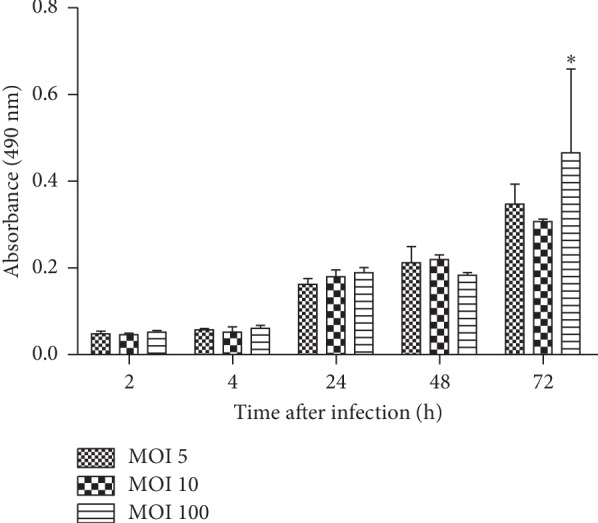
The LDH release from infected *D. discoideum*. Culture supernatants of infected *D. discoideum* cells were sampled and tested for LDH activity at 2, 4, 24, 48, and 72 h after infection. The LDH level was measured as absorbance values. The error bars represent standard deviations. ^*∗*^*p* < 0.05.

At 2 and 4 hours after infection, the LDH levels in supernatants of *D. discoideum* infected with *F. novicida* did not differ much between different MOI (2 h after infection with MOI 5 = 0.050, MOI 10 = 0.048, MOI 100 = 0.054; 4 h after infection with MOI 5 = 0.059, MOI 10 = 0.0535, MOI 100 = 0.0625) (Figure 3). At 24 and 48 hours after infection, *F. novicida* induced higher LDH release in comparison to the earlier time points, which is consistent with the results of the intracellular growth of *F. novicida* within *D. discoideum* (24 h after infection with MOI 5 = 0.163, MOI 10 = 0.180, MOI 100 = 0.190; 48 h after infection) with MOI 5 = 0.213, MOI 10 = 0.220, MOI 100 = 0.184. *F. novicida* induced the highest LDH release at 72 h after infection at MOI 100, in comparison to previous time points (74 h after infection with MOI 5 = 0.347, MOI 10 = 0.307, MOI 100 = 0.464) (Figure 3). The LDH release was not statistically different at observed time points with different MOI. Statistical difference was observed only at 72 hours after infection in comparison to cells infected with MOI 5 and MOI 100 (*p*=0.03), and MOI 10 with MOI 100 (*p*=0.01).

We can conclude that the increase of the LDH activity in the culture supernatant of the infected amoeba cells is proportional to the intracellular growth of bacteria within *D. discoideum*. Our results show that the MOI 10 induces the lowest level of cytotoxicity in *Dictyostelium*, so it will be used in our further experiments.

### 3.4. The Influence of Incubation Temperature on the Bacterial Growth within Amoeba

The influence of different incubation temperatures on the *F. novicida* growth within *D. discoideum* was also examined. Based on our previous results, the optimal dose of infection for the linear growth of bacteria within amoeba with low cytotoxicity is MOI of 10. Therefore, the amoeba cells were infected with MOI 10 and incubated at different temperatures (22°C, 25°C, 27°C, 30°C, and 37°C).

**Figure 4:**
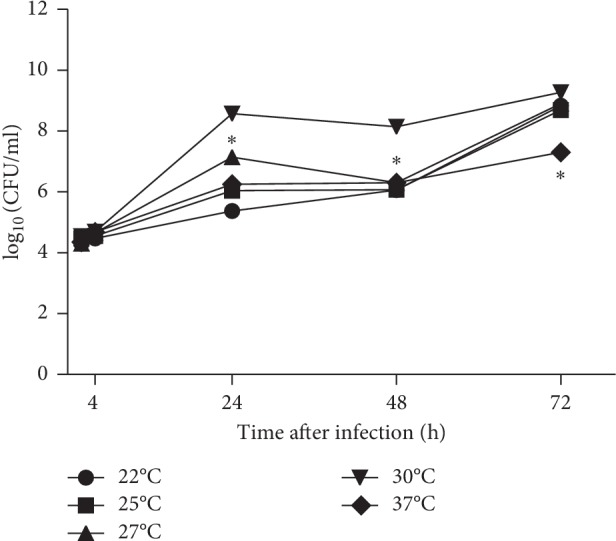
The growth kinetics of *F novicida* in *D. discoideum* after exposure to different temperatures. The *D. discoideum* cells were infected with *F. novicida* at MOI 10. The infected cells were incubated at 22°C, 25°C, 27°C, 30°C, and 37°C. At 2, 4, 24, 48, and 72 h after infection, the cells were lysed and plated on BCYE agar to determine the number of intracellular bacteria. The experiments were performed in triplicate. The error bars represent standard deviations. ^*∗*^*p* < 0.05.

At all observed temperatures, *F. novicida* showed similar intracellular growth at 2 and 4 hours after infection and the bacterial number increased up to 10^4^ CFU·mL^−1^. The bacterial cells continued to replicate within amoeba to 24 hours after infection. At 24 h after infection, the highest number of bacteria was determined at a temperature of 30°C (10^8^ CFU·mL^−1^) (Figure 4). In contrast, the lowest number of intracellular bacteria was observed 24 hours after infection at the incubation temperature of 22°C (10^5^ CFU·mL^−1^). At 72 hours after infection, the highest number of bacteria was determined after incubation on the temperature of 27 and 30°C (Figure 4).

Our results clearly show that the temperature of 30°C is an optimal temperature for *Francisella* intracellular growth within amoeba. In comparison with optimal temperature, the number of intracellular bacteria was statistically different at 24 hours after infection at 22°C (*p*=0.001), 25°C (*p*=0.001), 27°C (*p*=0.002), and 37°C (0.001). Also, the statistically different number of bacteria was found at 48 hours after infection at 22°C (*p*=0.001), 25°C (*p*=0.001), 27°C (*p*=0.002), and 37°C (0.002). At 72 hours after infection, only the number of intercellular bacteria observed after incubation of infected cells at 37°C was statistically different (*p*=0.001).

## 4. Discussion

Numerous studies have shown that the interaction between free-living amoeba and human pathogens, such as *F. tularensis*, *Legionella pneumophila*, and *Mycobacterium* spp., has significant implication in their environmental persistence. It was also shown that after growing within the amoeba, nonpathogenic bacteria gained their pathogenic features and that bacteria become more resistant to antibiotics [30–33]. In addition, many studies described amoeba like “Trojan horse” due to its protective role for bacteria in unfavorable environmental conditions and a role in the transmission of intracellular pathogens to humans [34, 35]. Many factors may play a role in the interaction of free-living amoeba and bacteria, such as incubation temperature and bacterial concentration. *Francisella tularensis* is a unique pathogen that can survive different environmental conditions and animal reservoirs. To be able to understand this wide range of hosts and the diversity of virulence strategies, there is a need for establishing more models for studying mechanisms required for infecting diverse hosts. This was successfully accomplished using *D. discoideum* as a host model to study the pathogenesis of *Klebsiella*, *Legionella*, *Pseudomonas*, and *Salmonella* infections [21–23, 36].

The effect of incubation temperature on the replication of Gram-negative bacteria *in vitro* was previously discussed in many researches. Although previous studies showed that the optimal temperature for studying the host-pathogen interaction within *Dictyostelium* cells is 21–23°C [21–23, 36], *Francisella* shows a little bit different behavior in comparison to other bacterial pathogens. The optimal growth of bacteria and *D. discoideum* was achieved at temperature 27–30°C. At lower temperatures, the growth of bacteria was significantly lower. Giving the concern that lower temperate may influence the expression of virulence traits, probably *F. novicida* somehow needs higher incubation temperature to be able to successfully replicate in this host. Our data show that *F. novicida* does not survive at 42°C, but it grows at 25 and 37°C. However, previous studies showed that subspecies *holartica* is more tolerant to a higher temperature (42°C) [8]. Each species of the genus *Francisella* requires different incubation temperatures. Various researches indicated that the findings of incubation temperature might improve the understanding of pathogenicity potential of the pathogens with the fastidious nature, such as *L. pneumophila* [37].

In addition, incubation temperature is crucial for the interaction between bacteria and amoeba. For the first time, in this study, we investigated the influence of incubation temperature on the replication of *F. novicida* within *D. discoideum*. The growth of bacteria within amoeba was temperature-dependent. Higher survival of *Francisella* within *Dictyostelium* was noted at a higher temperature (30°C) at the dose of infection 10. Due to *F. novicida* adaptation to environmental conditions, bacteria also have the ability to survive within amoeba at lower temperatures (22°C). In contrast, studies showed that *Legionella pneumophila* is unable to replicate intracellularly at room temperature, while it grew significantly within amoeba at 30, 32, and 37°C [38, 39]. Since it was shown that the growth of Gram-negative bacteria within amoeba depends on incubation temperatures, understanding the temperature dependence of *F. novicida* growth within amoeba will result in a better understanding of *F. novicida* ecological niches.

## 5. Conclusion

Our results demonstrate that the higher MOI (100) induce higher replication of *F. novicida* within amoeba with a high level of cytotoxicity. The optimal dose of infection is 10 at the incubation temperature of 30°C since it induces linear growth of *F. novicida* with low cytotoxicity in *Dictyostelium*.

Altogether, the survival and replication of *F. novicida* within amoeba depend on temperature and bacterial concentration. Since the free-living amoeba may be a reservoir for bacterial pathogens, growth factors in the interaction between bacteria and amoeba need to be clarified in future research for different *Francisella* subspecies.

## Data Availability

The Graph Pad data used to support the findings of this study are available from the corresponding author upon request.
